# Integrative transcriptomics and hypothesis-driven transfer machine learning reveal conserved and species-specific host-parasite dynamics across *Leishmania* species in THP-1 cells: a systematic review and meta-analysis

**DOI:** 10.3389/fcimb.2026.1891986

**Published:** 2026-08-26

**Authors:** Hawra Al-Ghafli, Aymen Alqurain, Faisal M. Alzahrani, Nasreldin Elhadi, Jignesh Prajapati, Haseeb Nisar

**Affiliations:** 1 Center for Biosystems and Machines, King Fahd University of Petroleum & Minerals (KFUPM), Dhahran, Saudi Arabia; 2 Bioengineering Department, King Fahd University of Petroleum Minerals (KFUPM), Dhahran, Saudi Arabia; 3 College of Pharmacy, Northern Border University, Rafha, Saudi Arabia; 4 Department of Clinical Laboratory Sciences, College of Applied Medical Sciences, Imam Abdulrahman Bin Faisal University, Dammam, Saudi Arabia; 5 Department of Biochemistry & Forensic Science, University School of Sciences, Gujarat University, Ahmedabad, Gujarat, India; 6 Center for Finance and Digital Economy, King Fahd University of Petroleum & Minerals (KFUPM), Dhahran, Saudi Arabia

**Keywords:** host-parasite interaction, integrative transcriptomics, *Leishmania*, systematic review and meta-analysis, THP-1 cells, transfer machine learning

## Abstract

**Introduction:**

Leishmaniasis is a vector-borne parasitic disease caused by protozoa of the genus *Leishmania*, characterized by clinical outcomes ranging from self-limiting cutaneous lesions to fatal visceral disease. Disease progression is multifactorial and is largely shaped by interactions between the parasite and host macrophages, as well as by other host- and pathogen-related factors. Although transcriptomic studies have provided insights into these interactions, differences in experimental design, parasite species, and analytical workflows have limited cross-study comparisons and the identification of conserved molecular responses.

**Methods:**

A systematic review was conducted following PRISMA guidelines to identify publicly available RNA-seq datasets of Leishmania-infected THP-1 macrophages. Raw sequencing data from eligible studies were reanalyzed using a unified bioinformatics pipeline incorporating standardized quality control, differential gene expression analysis, and batch-effect correction to enable robust cross-study integration. Comparative analyses were performed across infection stages and between *L. infantum* and *L. amazonensis*. The integrated transcriptomic dataset was subsequently used to develop a hypothesis-driven transfer learning framework to evaluate the feasibility of predicting *L. amazonensis* parasite gene expression at 96 hours post-infection (hpi) from experimentally generated 24 hpi transcriptomic profiles.

**Results:**

Integrated analysis revealed a pronounced early induction of pro-inflammatory and interferon-stimulated genes, including *CXCL10*, *IL1B*, and *IFIT1*, followed by attenuation of inflammatory signalling at later infection stages. Comparative analyses identified a conserved interferon-driven host response shared between *L. infantum* and *L. amazonensis*, together with species-specific transcriptional adaptations. The transfer learning framework demonstrated the feasibility of predicting late-stage parasite gene expression from early transcriptomic data, highlighting the potential of machine learning approaches to leverage limited transcriptomic datasets.

**Discussion:**

This study provides an integrated transcriptomic framework for investigating host-parasite interactions in *Leishmania*-infected macrophages and identifies conserved and species-specific transcriptional responses across infection. Furthermore, the proposed hypothesis-driven transfer learning approach demonstrates the potential to address transcriptomic data scarcity in neglected tropical disease research. Future *in vitro* studies and the availability of additional transcriptomic datasets will facilitate improved model training, validation, and generalizability.

## 1 Introduction

Leishmaniasis is a complex vector-borne parasitic disease caused by protozoa of the genus *Leishmania*. According to the World Health Organization (WHO), the disease affects millions of people worldwide, with 90,000 to 2 million new cases reported annually and an estimated 350 million people at risk of the infection (World Health Organization (WHO), 2010; Alvar et al., 2012; World Health Organization (WHO), 2023). Clinically, leishmaniasis presents a broad spectrum of diseases manifestations, ranging from self-healing cutaneous lesions to severe and often fatal visceral infections, which accounts for approximately 20,000–50,000 deaths each year (Alvar et al., 2012). This diversity in clinical outcome is largely driven by infection with different *Leishmania* species, including *L. major* and *L. amazonensis*, which are commonly associated with cutaneous leishmaniasis (CL), and *L. infantum*, a major causative agent of visceral leishmaniasis (VL) (World Health Organization (WHO), 2010; Kaye and Scott, 2011; Burza et al., 2018).

Following transmission by sandfly vectors, *Leishmania* parasites are rapidly internalized by host macrophages, where they differentiate into the intracellular amastigote form within a few hours of the infection and establish a specialized niche that supports long-term survival and replication (Olivier et al., 2005; Kaye and Scott, 2011). Thus, the clinical outcome of the infection is largely dictated by the ability of these amastigotes to persist within macrophages, the primary host immune cells targeted during infection (Kaye and Scott, 2011). This outcome reflects a dynamic interplay between parasite virulence mechanisms and host immune responses, which together determine whether the infection is cleared or progresses to a chronic disease (Sacks and Noben-Trauth, 2002). Despite extensive *in vitro* and *in vivo* investigations using diverse immune cell models and animal systems, the molecular mechanisms underlying host-parasite interactions remain incompletely resolved. In particular, discrepancies persist regarding the nature of protective versus pathogenic immune responses and the temporal dynamics that govern these outcomes across different host models and parasite species (Kwan et al., 1992; Buates and Matlashewski, 2001; Chaussabel et al., 2003; Gregory et al., 2008; Fortéa et al., 2009; Rabhi et al., 2012; Ramírez et al., 2012). For example, transcriptomic studies using RNA sequencing (RNA-seq) have provided valuable insights into macrophage responses to *Leishmania* infection, including immune activation, metabolic reprogramming, and parasite adaptation within host cells (Kwan et al., 1992; Chaussabel et al., 2003; Gregory et al., 2008; Rabhi et al., 2012; Ramírez et al., 2012). However, findings across studies are often difficult to reconcile due to heterogeneity in experimental design, parasite species, host cell lines, infection time points, and bioinformatic pipelines. Consequently, even key immune mediators such as *IL1B* have been reported as both protective and detrimental depending on the study context, underscoring the lack of consensus across studies (Lima-Junior et al., 2013; Novais et al., 2017). These inconsistencies limit the identification of conserved host-response signatures and hinder direct comparison of species-specific infection strategies.

Such inconsistencies are a recurring issue across high-throughput sequencing datasets, including cancer whole-exome sequencing studies, where data availability is generally not a limiting factor (Rasnic et al., 2019). In contrast, transcriptomic studies of host-parasite interactions, particularly in *in vitro Leishmania* infection models, are often constrained by limited sample sizes and heterogeneous experimental designs in addition to other biological variants. In this context, batch correction approaches developed for transcriptional data provide a critical opportunity to harmonize datasets across studies, reduce the impact of technical and experimental variability, and enable more robust identification of biologically conserved and species-specific host responses signals. Leveraging these approaches is particularly important for downstream integrative analyses and machine-learning models aimed at predicting gene expression dynamics to bridge the gap of limited data availability.

Among *in vitro* infection models, the THP-1 human monocytic cell line has emerged as a widely used system for studying macrophage-parasite interactions. THP-1-derived macrophages provide a reproducible and experimentally tractable model to investigate immune signaling, pathogen survival strategies, and transcriptional responses during infection (Daigneault et al., 2010; Chanput et al., 2014). In parallel, understanding species-specific differences among *Leishmania* parasites is essential, as distinct species exhibit substantial variation in pathogenicity, immune-modulation strategies, and disease outcomes (Burza et al., 2018). For instance, *L. infantum*, which causes VL, and *L. amazonensis*, a major cause of cutaneous disease, are known to induce divergent host responses and clinical phenotypes (Fernandes et al., 2023). However, comparative transcriptomic analyses of these species have largely been conducted in isolated studies that address different time points post infection, and often, by using different experimental conditions and analytical frameworks, limiting integrated interpretation of transcriptomic insights.

In this study, we address these challenges through a systematic meta-analysis of publicly available RNA-seq datasets generated from THP-1 macrophages infected with *Leishmania*. Raw sequencing data were analyzed using a unified bioinformatic pipeline that incorporates batch-effect correction to enable direct comparison of host transcriptional responses across studies and infection time points. Our work aims to: (i) define the core transcriptional signature of host macrophages during *Leishmania* infection, with particular emphasis on temporal changes in immune responses; (ii) identify conserved and species-specific host responses by comparing infections caused by *L. infantum* and *L. amazonensis* at early stages of the infection; and (iii) leverage the integrated transcriptomic dataset to explore the feasibility of a hypothesis-driven cross-species transfer learning framework for predicting late-stage gene expression profile and differentially expressed genes (DEGs) in *L. amazonensis*, for which experimental datasets remain limited.

Our findings reveal conserved immune responses, highlight species-specific transcriptional adaptations, and demonstrate the utility of machine-learning approaches for predicting parasite gene expression in infectious diseases. Together, this work provides a comprehensive framework that can be adapted to other host-parasite systems and offers an integrated foundation to support more *in vitro* studies and the identification of potential therapeutic targets for this neglected tropical disease.

## 2 Methodology

### 2.1 Systematic literature and screening

This systematic review was conducted in accordance with PRISMA guidelines (Moher et al., 2009). A comprehensive PubMed search was performed to identify RNA-seq studies of *Leishmania* infection in THP-1 cells using the query: ((*Leishmania*) AND (THP-1)) AND ((RNA-seq) OR (Transcriptome)), yielding 19 articles (**Supplementary Figure 1**). Studies were screened by two reviewers against predefined inclusion and exclusion criteria, retaining primary research using *in vitro* THP-1 models with publicly available RNA-seq data, clear experimental design, and publication within the last ten years (up to Sep 30, 2025). Reviews, secondary analyses, non-relevant sample types, and studies lacking sufficient methodological details were excluded. Eligible studies were further assessed by full-text review and summarized in a table including key experimental and sequencing information. Records were reviewed by two reviewers independently and discrepancy of inclusion conclusion was resolved by discussion prior to full text-review. In addition, studies lacking sufficient methodological details, those based on environmental or food-derived samples rather than *in vitro* infection models, publications outside the specified time frame, and articles published in languages other than English were excluded.

PubMed was used as the primary database to identify eligible peer-reviewed RNA-seq studies. GEO and SRA were not searched independently because they may contain duplicate submissions, datasets with limited methodological annotation, or records without an associated peer-reviewed publication, making them less suitable as the primary source for study identification in this systematic review. Instead, after eligible publications were identified, the corresponding raw RNA-seq datasets were located and retrieved from GEO/SRA for downstream transcriptomic meta-analysis.

### 2.2 Retrieval and processing of RNA-sequencing data

A total of 27 raw RNA-sequencing files were retrieved from public repositories (Gatto et al., 2020; Perea-Martínez et al., 2022; Fernandes et al., 2023) using the SRA Toolkit. Adapter sequences and low-quality bases were trimmed using Cutadapt (v1.2.1) with default parameters to ensure high-quality read retention. Clean reads were subsequently aligned to the human reference genome (GRCh38) using the STAR aligner (v2.5.2). In parallel, reads corresponding to the parasite were mapped to the *Leishmania* reference genomes: *L. infantum* JPCM5 (GCA_900500625.2) and *L. amazonensis* MHOM (GCA_025688915.1). Parasite genomic and annotation data were obtained from TriTrypDB (https://tritrypdb.org/tritrypdb/).

### 2.3 Differential gene expression and functional enrichment analysis

Raw read counts were summarized at the gene level, and count matrices from all samples were merged into a single dataset for both host and parasite analyses. To minimize technical variation, batch effects were corrected using the ComBat function from the sva R package (v3.54.0). Normalization and differential expression analyses were performed using edgeR. DEGs were identified based on a false discovery rate (FDR) < 0.05 and an absolute log_2_ fold change (|logFC|) ≥ 0.5.

Functional enrichment analysis of DEGs was conducted using Gene Ontology (GO) enrichment via the clusterProfiler (v4.14.6) and enrichplot packages in R (v1.26.6). Enriched GO terms were visualized through dot plots to highlight biological processes, molecular functions, and cellular components significantly affected by infections. Data visualization was performed using ggplot2 (v4.0.3), VennDiagram (v1.8.2), and heatmap.2, allowing the generation of expression heatmaps, volcano plots, and Venn diagrams between experimental conditions and time points. All analysis was done in R version 4.4.3.

Reactome over-representation analysis was performed for upregulated and downregulated DEGs (FDR < 0.05, logFC > 1) at each time point. The top 25 enriched pathways were compared across time points, and for common pathways, the number of identified entities was used to evaluate temporal changes in pathway enrichment.

### 2.4 Hypothesis-driven exploratory computational analyses

#### 2.4.1 Machine-learning for hypothesis-driven exploratory prediction of *L. amazonensis* gene expression at 96hpi

To explore whether cross-species transcriptomic information could be leveraged to address the scarcity of late-stage parasite transcriptomic datasets, we developed a hypothesis-driven exploratory transfer learning framework. The objective of this additional analysis was not to generate definitive predictions, but rather to evaluate the feasibility of predicting late-stage gene expression in *L. amazonensis* by exploiting conserved transcriptional patterns learned from related *Leishmania* species. Such an approach may provide a useful strategy for prioritizing candidate genes for future experimental validation while generating biologically testable hypotheses in data-limited settings.

Gene expression datasets for *L. infantum* and *L. amazonensis* were obtained from *in vitro* infection experiments included in this meta-analysis. The *L. infantum* dataset comprised five biological replicates at 24 hours post-infection (hpi) and three replicates at 96hpi, while *L. amazonensis* included five replicates at 24hpi. Expression values were normalized as log-transformed counts per million (logCPM). Orthologous gene pairs between species were identified using a curated orthology database, and only one-to-one matched genes were retained, with duplicates removed to ensure consistent feature mapping and resolved naming across both datasets.

To standardize sample size across datasets, the five *L. infantum* 24hpi replicates were averaged into three pseudo-replicates. These pseudo-replicates were generated through averaging operations rather than duplication of samples and were used solely to harmonize dimensional structure between datasets. Matched gene expression matrices were then constructed across *L. infantum* (24hpi and 96hpi) and *L. amazonensis* (24hpi) to maintain a shared feature space. Principal component analysis (PCA) was applied exclusively to the *L. infantum* training dataset at 24hpi and 96hpi for dimensionality reduction. The learned PCA transformation was subsequently applied to the corresponding *L. amazonensis* datasets. PCA fitting was performed only on the training species data, while the *L. amazonensis* dataset was transformed using the pretrained PCA space without refitting. This ensured that information from the evaluation dataset was not incorporated during feature learning or dimensionality reduction.

Additional feature augmentation was implemented only within the training dataset for the last set of models using controlled Gaussian noise injection to improve model robustness under limited sample conditions. Specifically, five noisy augmented duplicates per gene were generated in latent feature space (augment = 5) using low-amplitude Gaussian perturbation proportional to the standard deviation of each feature (noise_scale = 0.01). No augmentation, synthetic samples, or transformed derivatives from the evaluation/test datasets were introduced during training or model fitting, thereby preventing information leakage between training and test datasets.

Supervised machine-learning models-including Random Forest, Gradient Boosting, Support Vector Regression (SVR), Ridge Regression, Linear Regression, and Multi-Layer Perceptron (MLP)-were trained to predict *L. infantum* gene expression at 96hpi from 24hpi profiles. Model performance was evaluated using the coefficient of determination (R^2^). An 80/20 train-test split approach was employed to assess within-species accuracy of prediction on unseen features for *L. infantum*. Trained models were then applied to *L. amazonensis* 24hpi data to predict 96hpi expression based on ortholog mapping and retaining the same feature ID across the two species. Predicted expression profiles were compared with the nearest real observed data (i.e. transcriptomic gene expression dataset at 72hpi from human macrophage cells infected with *L. amazonensis*) using correlation analyses and R^2^ scores to assess temporal and cross-species predictive performance. Three sequential prediction frameworks were evaluated: (i) initial cross-species prediction models without augmentation or *L. major* integration, (ii) improved models incorporating training-data augmentation, and (iii) optimized models incorporating both augmentation and integrated *L. major* and *L. infantum* datasets during training. All analyses were performed in Python using the scikit-learn library.

#### 2.4.2 MHC class II binding and epitopes identification

To explore one potential application of the hypothesis-driven transfer learning framework, we performed *in silico* epitope prediction on a subset of predicted DEGs. This analysis was intended to prioritize candidate parasite proteins for future experimental validation rather than to establish definitive antigenic targets. Three candidate genes were selected from the predicted dataset, and notably, two were also independently identified as differentially expressed in the nearest available experimental *L. amazonensis* transcriptomic dataset (72hpi vs. 24hpi), providing additional support for the hypothesis-driven predictions.

Protein sequences corresponding to these three DEGs from *L. amazonensis* were retrieved from TriTrypDB and compiled in FASTA format-corresponding to hypothetical proteins of unknown function. Prediction of CD4^+^ T-cell epitopes was performed using the IEDB Analysis Resource, applying the NetMHCIIpan EL algorithm under default recommended settings (Dhanda et al., 2019). Peptide length was set to 15 amino acids, consistent with typical MHC class II binding preferences. A representative panel of all human HLA class II alleles was used. Predicted peptides were ranked based on percentile scores, where lower values indicate stronger binding affinity. Peptides with a percentile rank of less than 0.5 were considered strong binders and selected for downstream analysis. To reduce redundancy, overlapping peptides sharing the same core binding region were grouped, and a single representative peptide with the lowest percentile rank was retained. Regions generating multiple high affinity overlapping peptides were identified as putative immunodominant regions. Selected high-affinity, non-redundant peptides were subsequently prepared for structural analysis and molecular docking with corresponding MHC class II molecules. Structural data for MHC molecules were obtained from the Protein Data Bank (PDB), including structures 4D8P, 1BX2, and 1AQD, and peptides were curated to ensure compatibility with docking workflows.

#### 2.4.3 Computational analysis of peptide structure and protein-peptide interactions of three *L. amazonensis* DEGs

Four peptides were selected for *in silico* structural and functional characterization based on prior analyses. Secondary structure prediction was performed using Chou–Fasman and GOR IV methods, classifying residues into α-helix, β-strand, or coil. Physicochemical properties, including molecular weight, isoelectric point, net charge, GRAVY score, aliphatic index, and instability index, were calculated using standard bioinformatic approaches. Hydrophobicity profiling (Kyte–Doolittle scale), intrinsic disorder prediction, and functional motif identification were conducted to assess structural features and potential biological roles. For structural interaction analysis, protein-peptide docking was performed using ClusPro 2.0, with HLA receptor structures obtained from the PDB. The most energetically favorable docking models were selected based on lowest and central pose energy values, and interaction analysis was carried out using BIOVIA Discovery Studio Visualizer.

## 3 Results

### 3.1 Integration and quality assessment of transcriptomic datasets

One of the main objectives of this study is to gather and reanalyze all existing RNA-seq data to identify key host-parasite transcriptomic signatures within THP-1 cell line in response to *L. infantum* infection and resolve discrepancies in insights (Fernandes et al., 2016; Gatto et al., 2020; Perea-Martínez et al., 2022; Fernandes et al., 2023). To ensure comparability across experiments, all RNA-seq datasets included in this study were processed using a unified analysis pipeline (**Table 1**). Raw sequencing reads from each included study were mapped to the same reference genome, and gene expression counts were generated using identical alignment and quantification parameters. Because the datasets originated from multiple experiments and time points, batch effects were corrected prior to downstream analysis to minimize technical variability and enable accurate comparison of transcriptional profiles across conditions.

**Table 1:** Study characteristics of included studies and dataset in this paper.

Study ID in figures	Study title and year	Time of infection	Target species	Parasite species	Sequencing	SRA_ID	Bioinformatics pipeline in the original study	Included raw sequencing files	Purpose of inclusion
m4	Transcriptional analysis of THP-1 cells infected with *Leishmania infantum* indicates no activation of the inflammasome platform. (2020)	8hpi	Host & parasite	*Leishmania infantum*	Single-end reads, (Illumina) NextSeq 500 Platform	PRJNA552352	Trimmomatic v0.36, HISAT, the *Leishmania infantum* genome (GCA_000002875.2), Homo sapiens genome (GRCh38, gencodev24), BEDTools intersect v2.25.0, and DESeq2 for DEGs	6 raw files (SRR9624224, SRR9624225, SRR9624226, SRR9624227, SRR9624230, SRR9624231)	Machine-learning and meta-analysis
m5	Comparative transcriptomic analysis of long noncoding RNAs in *Leishmania-*infected human macrophages. (2023)	24hpi	Host	*Leishmania amazonensis*, *Leishmania braziliensi, Leishmania infantum*	Paired-end reads, (Illumina) Novaseq 6,000 Platform	PRJNA881925	FastQC tool, human reference genome assembly (GRCh38) was done using bowtie2, and edgeR for DEGs	15 raw files (SRR21631095, SRR21631100,SRR21631105, SRR21631096, SRR21631101, SRR21631106, SRR21631097, SRR21631102, SRR21631107, SRR21631098, SRR21631103, SRR21631113, SRR21631099, SRR21631104, SRR21631114)	Machine-learning and meta-analysis
m6	Transcriptomic analysis in human macrophages infected with therapeutic failure clinical isolates of *Leishmania infantum*. (2022)	96hpi	Host & parasite	*Leishmania infantum*	Paired-end, reads (Illumina) NextSeq 500 Platform	PRJNA836366/PRJNA781438	FastQC software, Seqtk software, HISAT2, Homo_sapiens. GRCh38 as reference genome and edgR for DEGs	6 raw files (SRR16976280, SRR16976281, SRR16976282, SRR16976283, SRR16976284, SRR16976285)	Machine-learning and meta-analysis
NA	Dual Transcriptome Profiling of *Leishmania-Infected* Human Macrophages Reveals Distinct Reprogramming Signatures (2016)	4hpi, 24hpi, 48hpi and 72hpi	Host & parasite	*Leishmania amazonensis, Leishmania major*	Paired-end reads, (Illumina) HiSeq 1500 platform.	PRJNA290995, PRJNA252769, and PRJNA292915.	Trimmomatic, TopHat, HTSeq, *L. amazonensis*-infected samples were aligned to *L. mexicana* (v. 8.1) genome	21 raw files (SRR2163251, SRR2155160, SRR2156107, SRR2156274, SRR2163242, SRR2163283, SRR2156117, SRR2156271, SRR2156856, SRR2163272, SRR2163276, SRR2163279, SRR2163289, SRR2163399, SRR2155085, SRR2155160, SRR2156107, SRR2156117, SRR2156860, SRR2163251, SRR2163285),	Improve training for Machine-learning using 24hpi and 72hpi RNA-seq sequencing files for *Leishmania amazonensis and Leishmania major*

**Figure 1:**
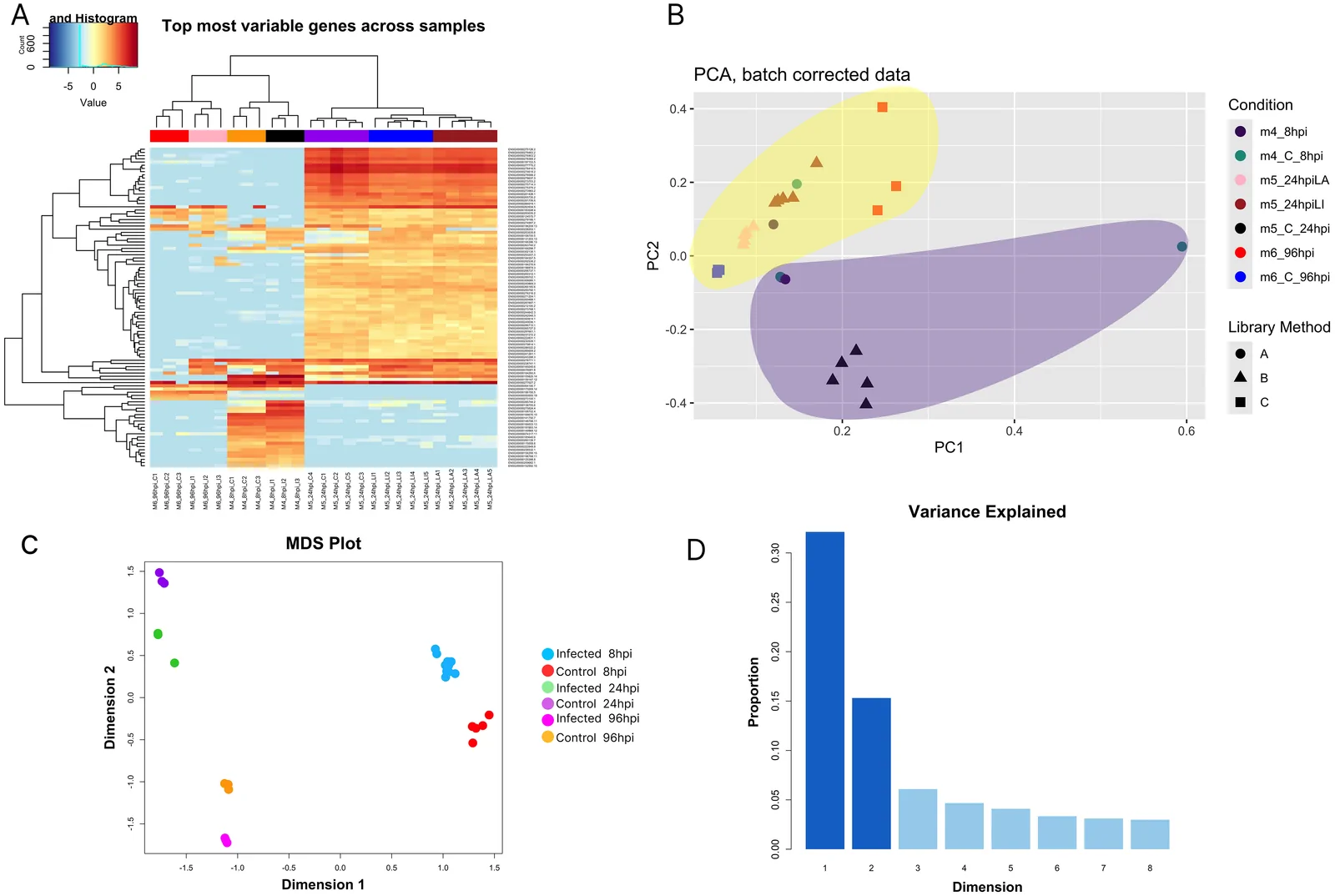
Dataset integration and clustering of THP-1 transcriptomic responses to *Leishmania* infection. **(A)** Heatmap of the most variable genes across samples showing hierarchical clustering of infected and control conditions after batch correction. Red color indicate relatively higher expression and blue indicating relatively lower expression **(B)** Principal component analysis (PCA) of batch-corrected expression profiles showing separation of infected and control samples. Library/study labels correspond to A = m4 = Gatto et al. (2020); B = m5 = Fernandes et al. (2023); and C = m6 = Perea-Martínez et al. (2022). While different colors denote the experimental conditions within each study **(C)** Multidimensional scaling (MDS) plot illustrating clustering of samples by infection status and time point. **(D)** Scree plot showing the proportion of variance explained by each principal component for the MDS plot.

A heatmap of the most variable genes revealed clear segregation between infected and control samples, indicating strong time and infection-associated transcriptional changes in THP-1 cells (**Figure 1A**). Hierarchical clustering showed that infected samples grouped separately from controls, with additional clustering by infection time point (**Figure 1A**). PCA of the batch-corrected dataset further confirmed this separation, with infected and control samples clearly distinguished along the first component (**Figure 1B**). Compared with the uncorrected dataset (**Supplementary Figure 2**), batch correction reduced the apparent influence of batch effects and improved the separation of samples according to infection status. Multidimensional scaling (MDS) analysis showed tight clustering of replicates within each condition, indicating high consistency across datasets, with separation between infected and non-infected datasets becoming more pronounced at 8h, followed by 24h, and then 96h (**Figure 1C**), suggesting that as the infection progresses, there is an extensive immune-modulation in which the cell transcriptomic profile becomes more similar to control cells. The first two principal components accounted for most of the variance in the combined transcriptomic dataset, whereas the remaining components each explained only a small proportion of the total variance (**Figure 1D**).

### 3.2 Dynamic differential gene expression analysis in host THP-1 cells reveals progressive modulation of immune processes

**Figure 2:**
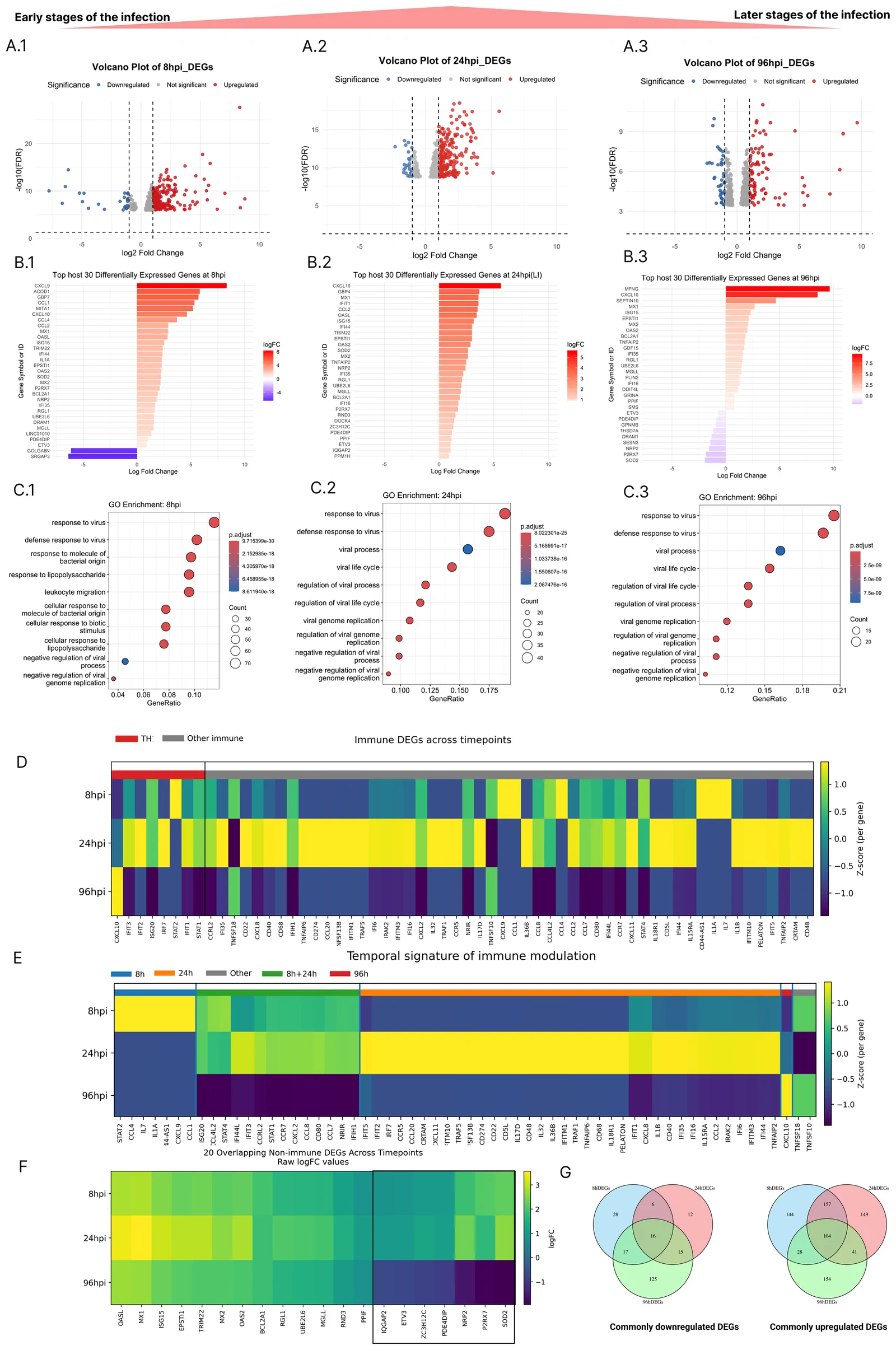
Dynamic differential gene expression in THP-1 cells infected with *Leishmania*. **(A)** Volcano Plots illustrating the global differential gene expression (DEG) profile of host THP-1 cells at 8 hours post-infection (hpi), 24hpi, 24 and 96hpi (A.1, A.2 and A3). Red indicates significantly upregulated genes, blue indicates significantly downregulated genes, and grey indicates non-significant genes. **(B)** shows top 30 upregulated DEGs at each time point, ranked by magnitude of logFC at 8hpi, 24hpi, and 96hpi in (B.1–B.3). **(C)** illustrates GO enrichment of DEGs showing key biological processes for each prospective time point (C.1–C.3). Bubble size represents the number of genes associated with each GO term; color represents adjusted *p-*value. **(D)** A heatmap of immune-related differentially expressed genes (DEGs) across time points is shown, with Z-scores of relative gene expression used to highlight Th1-related genes in comparison to other immune genes. **(E)** Temporal clustering of immune genes across time points (Z-scores of relative gene expression) highlighting distinct expression patterns across infection stages. **(F)** Heatmap of overlapping non-immune DEGs shared across time points (logFC). **(G)** Venn diagrams showing overlap of commonly upregulated and downregulated DEGs across time points (infected vs control).

We then sought to examine DEGs across different time points. Our combined analysis reveals a progressive shift in host transcriptional responses across infection time points (**Figures 2A.1–A.3**), with a noticeable reduction in the number and magnitude of upregulated genes as infection proceeds from early (8hpi) to late stages (96hpi). At 8hpi, a robust transcriptional response was evident, characterized by a large proportion of significantly upregulated genes. This response diminished at 24hpi and was further attenuated by 96hpi.

Examination of the top DEGs for each comparison (infected vs. control) highlighted a set of key immune-related genes that were strongly induced early but declined progressively across time points (**Figures 2B.1–B.3, D**). These genes were predominantly associated with pro-inflammatory and Th1-associated immune signaling pathways, among other chemokines and interferon-responsive regulators such as *CXCL10*, *STAT1*, *IL1B*, and *TNF*. While some of these genes remained detectable at later stages, their expression levels and relative ranking among top DEGs were reduced. GO enrichment analysis supported these observations (**Figures 2C.1–C.3**), with early time points enriched for processes related to response to virus, leukocyte migration, chemotaxis, and response to bacterial molecules, reflecting strong innate inflammatory activation. At 24hpi, however, enrichment remained centered on immune cell recruitment and migration pathways, whereas by 96hpi, the profile shifted toward broader immune and regulatory processes, including comparatively lower modulation of antiviral and cellular defense responses relative to earlier infection stages.

To further characterize temporal regulatory patterns, relative gene expression of immune-related DEGs across time points was compared (**Figures 2D, E**). This analysis revealed a clear trend of gradual downregulation for a substantial subset of immune-related genes, particularly those associated with Th1 responses following their early induction. Conversely, a smaller subset of genes exhibited sustained or late-stage pronounced relative expression, suggesting a transition from an acute inflammatory response toward a more immune-regulated host state. Overlap analysis further identified a core set of DEGs shared across time points (**Figures 2F, G**), representing conserved host responses to *L. infantum*. Our findings demonstrate that *L. infantum* infection elicits a strong early pro-inflammatory and Th1-associated transcriptional response that is progressively attenuated, accompanied by a shift toward broader immune regulatory programs, consistent with the establishment of a controlled or persistent infection state.

These temporal trends were further supported by Reactome pathway analysis (**Tables 2**, **3**). Among pathways shared across all time points, immune-related enrichment was strongest at 8–24hpi and attenuated by 96hpi, as indicated by fewer upregulated genes contributing to major interferon-, cytokine-, and interleukin-signaling pathways. In contrast, downregulated DEGs showed a temporal shift from enrichment of metabolic and epigenetic pathways at early time points to suppression of IL-12/JAK–STAT and cytokine signaling pathways at 96hpi, consistent with progressive immune-modulation during infection.

**Table 2:** Comparison of common significantly enriched Reactome pathways among upregulated DEGs (logFC > 1, FDR < 0.05) across infection time points.

Reactome pathway	8hpi entities	24hpi entities	96hpi entities	Interpretation
Interferon Signaling	75	76	47	Markedly reduced at 96hpi
Cytokine Signaling in Immune System	123	109	66	Progressive reduction
Immune System	142	130	72	Progressive reduction
Interferon alpha/beta signaling	45	47	29	Reduced at 96hpi
Interferon gamma signaling	29	29	15	Approximately 50% reduction
Antimicrobial mechanism of IFN-stimulated genes	28	29	21	Moderately reduced
Interleukin-10 signaling	27	20	10	Strong reduction
Signaling by Interleukins	53	38	19	Progressive reduction
Interleukin-4 and Interleukin-13 signaling	21	14	14	Early decline then stable
IFIT antiviral response	10	10	7	Mild reduction
ISG15 antiviral mechanism	9	10	5	Approximately 50% reduction
Non-canonical inflammasome activation	6	5	5	Largely maintained
ATF4 activates genes in response to ER stress	6	6	4	Mild reduction
PERK regulates gene expression	6	6	4	Mild reduction
OAS antiviral response	4	5	3	Mild reduction
CLEC7A/inflammasome pathway	3	3	3	Maintained

**Table 3:** Comparison of enriched Reactome pathways among downregulated DEGs (logFC < –1, FDR < 0.05).

Reactome pathway	8hpi	24hpi	96hpi	Pattern
Differentiation of Circulating Monocytes	✓	✓	—	Early/intermediate only
TP53 Regulates Metabolic Genes	✓	✓	—	Early/intermediate only
Epigenetic regulation of gene expression	✓	✓	—	Early/intermediate only
Lipophagy	✓	✓	—	Early/intermediate only
Inactivation of CDC42 and RAC1	✓	✓	—	Early/intermediate only
PPARA activates gene expression	✓	✓	—	Early/intermediate only
Regulation of lipid metabolism by PPARα	✓	✓	—	Early/intermediate only
Chaperone-mediated autophagy	✓	✓	—	Early/intermediate only
RAC1 GTPase cycle	✓	✓	—	Early/intermediate only
Gene/protein expression by JAK–STAT after IL-12 stimulation	—	—	✓	Late only
Interleukin-12 signaling	—	—	✓	Late only
Interleukin-12 family signaling	—	—	✓	Late only
Signaling by Interleukins	—	—	✓	Late only
Cytokine Signaling in Immune System	—	—	✓	Late only
Interleukin-20 family signaling	—	—	✓	Late only

### 3.3 Comparative analysis of species-specific host response at 24hpi

**Figure 3:**
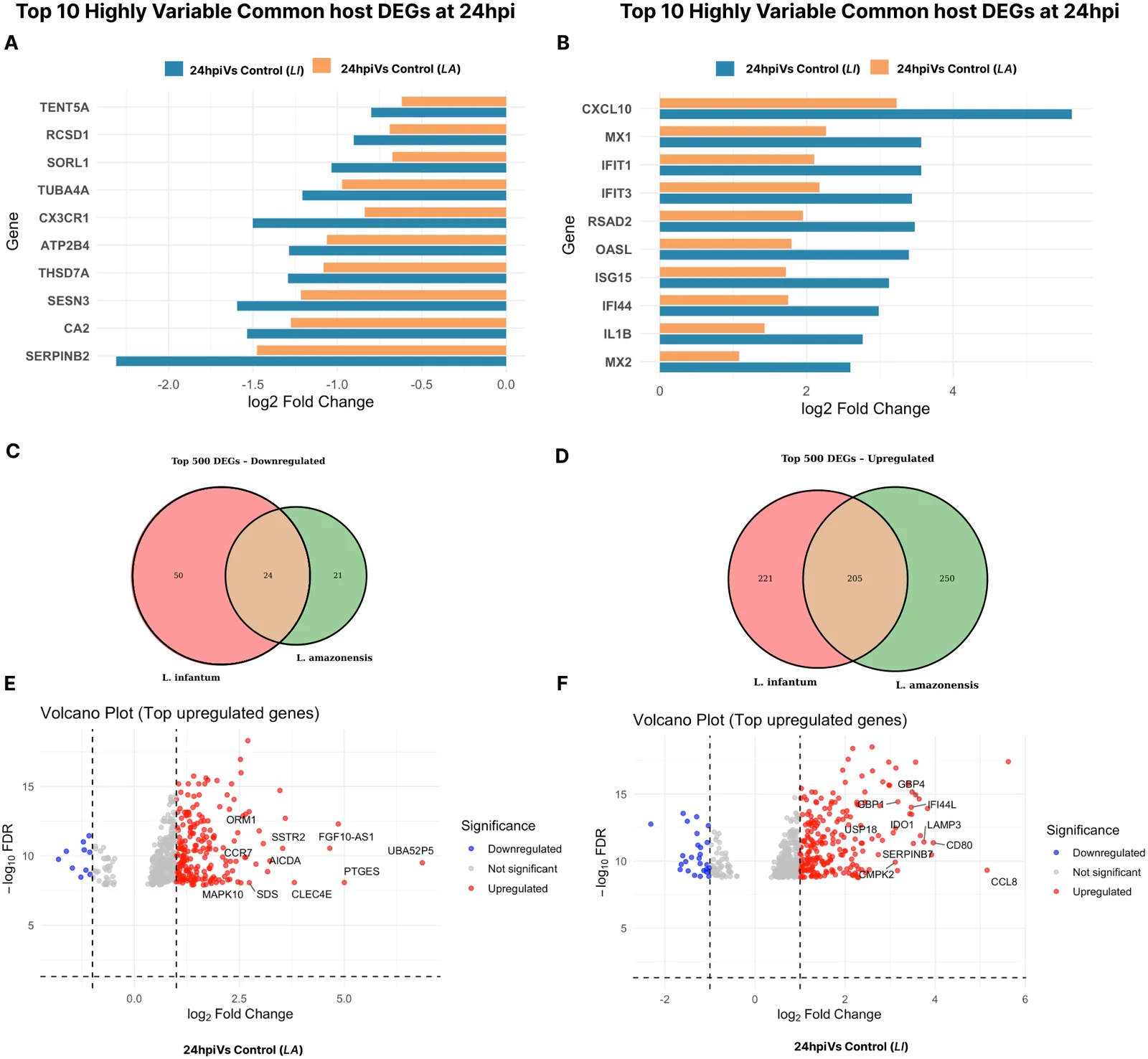
Comparative host transcriptional responses to *L. infantum* and *L. amazonensis* infection at 24hpi. **(A)** Bar plot showing the top 10 most variable commonly downregulated host genes at 24hpi in *L. amazonensis* (LA) and *L. infantum* (LI) infections compared with control samples. **(B)** Bar plot showing the top 10 most variable commonly upregulated host genes shared between LA and LI infections. Venn diagram illustrating overlap of host DEGs in response to either LA and LI infection **(C, D)**. Volcano plots **(E, F)** display global differential gene expression profiles in infected THP-1 cells compared with controls highlighting some uniquely upregulated DEGs for each infection.

To compare host responses induced by different *Leishmania* species in the same model system, differential gene expression profiles at 24hpi were analyzed for *L. infantum* and *L. amazonensis* infections (**Figure 3**). Analysis of the most variable host genes revealed both shared and species-specific transcriptional responses. A set of genes was consistently downregulated in both infections, including *TENT5A, RCSD1, SORL1, TUBA4A*, and *CX3CR1*, suggesting common suppression of certain host cellular processes during infection (**Figure 3A**). In contrast, a group of genes showed strong and consistent upregulation across both infections, including *CXCL10, MX1, IFIT1, IFIT3, RSAD2, OASL, ISG15, IFI44, IL1B*, and *MX2* (**Figure 3B**). Many of these genes are interferon-stimulated genes (ISGs) and key mediators of inflammatory and antiviral responses, indicating a consistent pattern of transcriptomic signatures of host responses during 24hpi for these two *leishmania* species.

205 upregulated genes were common to both *L. infantum* and *L. amazonensis* infections, whereas 221 genes were uniquely upregulated in *L. infantum* infection and 250 genes were uniquely upregulated in *L. amazonensis* infection (**Figures 3C, D**). Uniquely highly induced genes included inflammatory mediators and some interferon-responsive genes such as *SERPINB2*, *LAMP3*, *IFI44L*, *GBP4*, and *CCL8*, highlighting activation of immune defense pathways in infected cells by *L. infantum* (**Figure 3F**), whereas uniquely highly induced top DEGs due to *L. amazonensis* infection include *UBA52P5*, *PTGES* and *MAPK10* (**Figure 3E**). Together, these results demonstrate that while *L. infantum* and *L. amazonensis* infections trigger a shared transcriptional host response, they also exhibit some level of species-specific differences in host gene regulation.

### 3.4 Hypothesis-driven cross-species prediction of gene expression using transfer learning

Considering that a conserved host immune transcriptional response was observed at 24hpi for both *L. amazonensis* and *L. infantum*, and given that a 96hpi gene expression profile for *L. amazonensis* is not currently available, we sought to predict the late-stage transcriptional profile of *L. amazonensis* via transfer machine-learning. This approach was designed to enable a comparative analysis of parasite transcriptional dynamics between the two species at later stages of infection, where host responses are more profoundly suppressed, to infer if this is mediated via a conserved parasite mechanism of infection.

**Figure 4:**
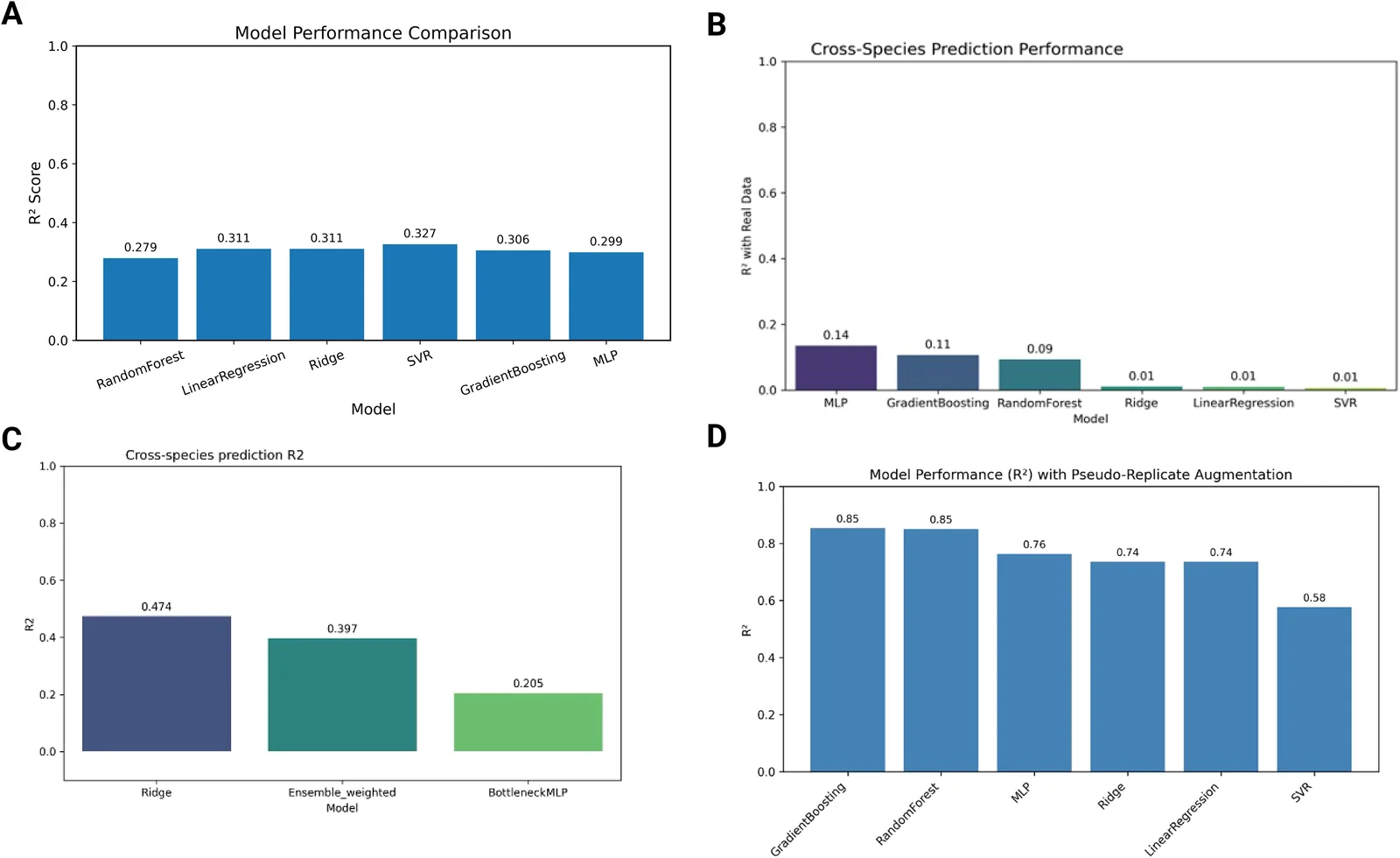
Transfer machine-learning framework for cross-species prediction of *Leishmania* gene expression dynamics. **(A)** Within-species model performance for predicting *L. infantum* gene expression at 96hpi from 24hpi data using multiple machine-learning algorithms. Models were initially trained with 80% of the 8,736 ortholog-matched features between the two species, and their within-species prediction was evaluated using 20% of the unseen features. **(B)** Shows cross-species prediction performance, where models initially trained on *L. infantum* data and then used to predict *L. amazonensis* expression at 96hpi, showing limited generalization across species. **(C)** Improved cross-species prediction using dimensionality reduction and transfer learning approaches, highlighting enhanced performance compared to baseline models trained on *L. infantum* alone. **(D)** Optimized transfer learning framework incorporating multi-species training data (*L. infantum* and *L. major*) and pseudo-replicate augmentation on the training dataset only (7,353 ortholog-matched features across the three species), resulting in substantially improved prediction accuracy across models compared to the nearest real transcriptomic data for *L. amazonensis-*human macrophages at 72hpi.

To achieve this, multiple machine-learning models were trained on temporal gene expression data from *L. infantum*, utilizing matched 24hpi and 96hpi gene expression datasets. Models initially learned within-species temporal relationships, showing moderate predictive performance but limited generalization (R^2^ = 0.27), indicative of overfitting and reduced capacity to capture nonlinear dynamics of unseen features (**Figure 4A**). When extended to cross-species prediction, in which we tried to infer 96hpi expression profile for *L. amazonensis* from 24hpi data, models performance declined substantially (R^2^ ≤ 0.14), highlighting constraints imposed by species-specific regulatory differences despite ortholog-based feature alignment (**Figure 4B**). To address these limitations, we implemented a transfer learning strategy incorporating dimensionality reduction and latent space representation. A PCA-based temporal transfer model combined with ridge regression improved cross-species prediction (R^2^ = 0.47), suggesting that latent feature compression enhances conservation of transcriptional signals across species (**Figure 4C**).

Further improvement was achieved by integrating multi-species datasets (datasets on both *L. infantum* and *L. major*) and applying small scale pseudo-replicate augmentation, which substantially enhanced predictive performance. Under this optimized framework, gradient boosting and random forest models achieved the highest accuracy (R^2^ = 0.85), followed by MLP and linear models (R^2^ ≈ 0.74–0.76), demonstrating that increased data diversity and effective sample size representation improve significantly cross-species generalization (**Figure 4D**). These findings highlight the potential of transfer learning approaches to substantially improve cross-species prediction of gene expression dynamics in *Leishmania*-THP-1 studies in the future. Such strategies offer an adaptable solution to data scarcity and may further advance our understanding of comparative parasite adaptation mechanisms with limited resources.

### 3.5 Comparison of temporal gene expression dynamics between *L. infantum* and predicted *L. amazonensis*

**Figure 5:**
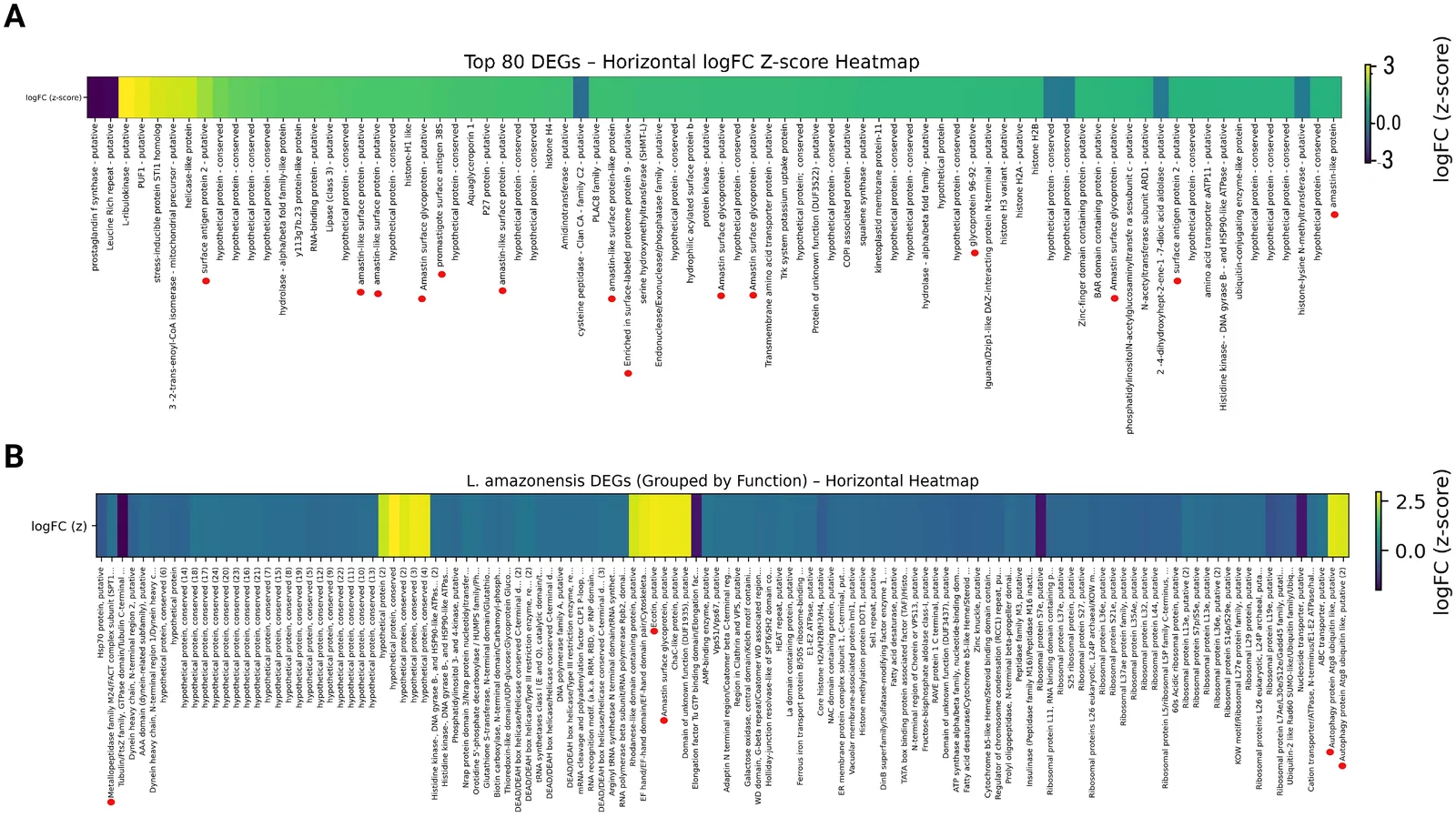
Parasite differential gene expression between early and late infection stages. **(A)** Heatmap of experimentally measured differential gene expression (logFC, row Z-score) in *L. infantum* comparing 96hpi to 24hpi, highlighting genes upregulated or downregulated during late infection. **(B)** Heatmap of machine-learning-predicted differential gene expression in *L. amazonensis* (96hpi vs 24hpi), grouped by functional gene categories. Colors represent scaled log fold-change values, illustrating predicted transcriptional dynamics across parasite genes, with yellow indicating relative upregulation and blue indicating relative downregulation.

Differential gene expression analysis revealed pronounced transcriptional remodeling in *L. infantum* between early (24hpi) and late (96hpi) infection stages. The top 80 DEGs, visualized as a horizontal heatmap, showed a predominance of upregulated transcripts at 96hpi relative to 24hpi (**Figure 5A**). These included multiple genes annotated as hypothetical proteins, as well as factors associated with protein synthesis, surface antigen expression, and metabolic processes. In contrast, a smaller subset of genes exhibited strong relative downregulation, suggesting selective suppression of pathways potentially linked to parasite adaptation and persistence during late-stage infection. Overall, the transcriptional profile indicates a shift toward a more targeted and specialized state as infection progresses.

Using the trained transfer learning framework, predicted differential expression profiles for *L. amazonensis* revealed comparable but distinct transcriptional patterns compared to *L. infantum* (**Figure 5B**). Most genes displayed moderate relative downregulation, consistent with a conserved trend of transcriptional suppression during late infection. However, discrete clusters of strongly upregulated genes were observed, particularly within specific functional categories, including stress response, protein turnover, and ribosomal components. Together, these results support partial conservation of temporal transcriptional responses across *Leishmania* species while also revealing species-specific regulatory divergence.

### 3.6 Exploratory *in silico* characterization of candidate MHC Class II-binding peptides

Prediction of epitopes was performed for protein sequences corresponding to three *L. amazonensis* DEGs (*LAMA_000019500*, *LAMA_000051800*, and *LAMA_000215500*). Two of these DEGs were identified in both the hypothesis-driven transfer learning predictions and the nearest available experimental 72hpi transcriptomic dataset (labelled as prot2 and prot3). The four peptides displayed diverse physicochemical and structural properties, spanning hydrophobic to hydrophilic profiles, variable charge states, and distinct secondary structure tendencies, as summarized in **Supplementary Table 1**. Structural predictions revealed β-sheet dominance, α-helical enrichment, intrinsic disorder, or mixed conformations depending on peptide composition, alongside functional motifs associated with membrane interaction, nucleic acid binding, or structural flexibility.

**Figure 6:**
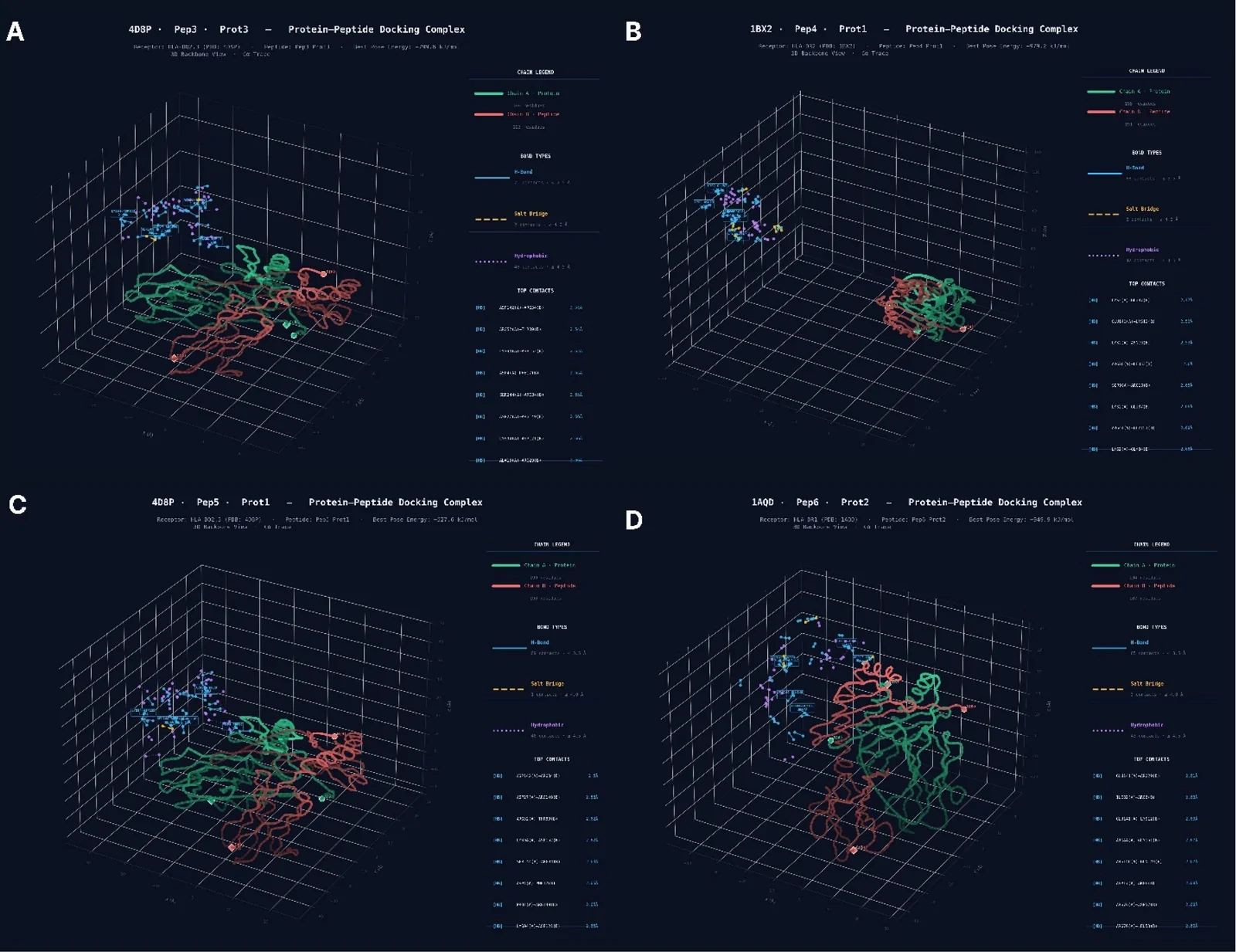
Structural docking analysis of predicted MHC class II–binding peptides derived from three *Leishmania amazonensis* DEGs proteins. Docking models showing interactions between predicted high-affinity peptides and MHC class II receptor structures. Panels **(A–D)** represent four peptide–protein complexes: **(A)** 4D8P–Pep3–Prot3, **(B)** 1BX2–Pep4–Prot1, **(C)** 4D8P–Pep5–Prot1, and **(D)** 1AQD–Pep6–Prot2. Peptides are shown in cyan and receptor proteins in green, with interaction sites highlighted. The complexes demonstrate stable binding with key interactions including hydrogen bonds, salt bridges, and hydrophobic contacts, supporting the potential of these peptides for MHC class II presentation.

Docking analysis demonstrated stable binding of all peptides to HLA receptors, with favorable energy profiles (**Supplementary Table 2**) and interaction patterns characterized by hydrogen bonds, salt bridges, hydrophobic contacts, and π-π stacking interactions (**Supplementary Table 3**, **Figure 6**). Representative docking complexes are shown in **Figures 6A–D**, illustrating peptide positioning within the receptor binding interface and highlighting distinct interaction networks across complexes. Notably, complexes such as 1BX2_Pep4_Prot1 and 4D8P_Pep5_Prot1 exhibited extensive interaction networks, indicating strong binding affinity and specificity, while others showed balanced electrostatic and hydrophobic contributions. Overall, these findings highlight structurally and functionally distinct peptides with stable receptor-binding capabilities, supporting their potential biological relevance.

## 4 Discussion

Despite extensive investigation of macrophage responses to *leishmania* infection, existing evidence remains partially inconsistent, with studies reporting divergent immune phenotypes ranging from robust pro-inflammatory activation to immune suppression or regulatory polarization at different time-points by different *leishmania* species. It remains challenging to disentangle the extent to which these discrepancies arise due to biological variation versus technical heterogeneity across experimental systems and analytical pipeline. The temporal patterns observed in the present study reconcile some of these discrepancies by integrating publicly available RNA-seq datasets generated from THP-1 cells infected with either *L. infantum* or *L. amazonensis* and analyzing them through a unified analytical pipeline with appropriate batch correction to allow us to establish a temporal understanding of transcriptomic changes associated with infection. Specifically, the early induction of inflammatory mediators and interferon-stimulated genes in THP-1 cells-such as *CXCL10*, *IL1B*, *TNF*, and *ISG15*-is consistent with reports describing an initial M1-like activation phase following infection (Lima-Junior et al., 2013; Gurung and Kanneganti, 2015). This is accompanied by a relative enrichment of chemokines associated with M1 polarization, including *CXCL2*, *CXCL8*, *CXCL9*, *CCL2*, and *CCL4* (**Figure 2E**), that play a critical role in active recruitment of various T and immune cells during *leishmania* infection (Tomiotto-Pellissier et al., 2018; Elmahallawy et al., 2021). However, the subsequent attenuation of these responses at later time points aligns with studies demonstrating that *L. infantum* can actively reprogram macrophages toward a more permissive or regulated state that does not conform strictly to the classical M1/M2 paradigm (Elmahallawy et al., 2021). such transcriptional temporal shift may therefore explain why some studies identify a predominant inflammatory profile, often linked to high nitric oxide (NO) production, whereas other studies report suppressed or mixed phenotypes, depending on the stage of infection examined *in vitro* and *in vivo* (Miralles et al., 1994; Vellozo et al., 2017; Omar and Abdelal, 2022).

Furthermore, *IL1B* is strongly induced at early time points and remains elevated at 24hpi (**Figure 2**), indicating a sustained early inflammatory response. However, the role of IL1B in VL remains incompletely resolved. On one hand, IL1B has been implicated in protective immunity, where pro-inflammatory cytokine responses contribute to macrophage activation and parasite control (Lima-Junior et al., 2013; Gurung and Kanneganti, 2015). On the other hand, multiple studies in human VL caused by *L. infantum* have reported elevated systemic levels of IL1B in active disease, which correlate with disease severity rather than protection, suggesting a role in pathology and immune dysregulation (Faleiro et al., 2014; Santos et al., 2018; Margaroni et al., 2022). For instance, increased IL1B concentrations have been consistently detected in VL patient sera compared to healthy controls, supporting its association with active infection and inflammatory burden rather than effective clearance (Babaloo et al., 2020). Moreover, additional cytokine profiling studies in VL indicate that increased inflammatory responses, including IL1β and other pro-inflammatory mediators, can occur in parallel with impaired parasite control, contributing to systemic inflammation and disease progression (dos Santos et al., 2016; Santos et al., 2018; Margaroni et al., 2022). These findings position IL1β as a temporally regulated mediator, in which its contribution to protection versus pathology in *L. infantum* infection is dependent on infection stage and the balance between pro-inflammatory and regulatory immune pathways.

Recent advances in cross-species machine-learning have demonstrated that transcriptional profiles can be learned from one biological system and successfully transferred to another, enabling the identification of conserved disease mechanisms (Normand et al., 2018; Taroni et al., 2019; Cheng et al., 2024). This approach is particularly valuable in the context of neglected tropical diseases, where experimental resources are often limited and generating comprehensive datasets across multiple *Leishmania* species is challenging (Banerjee et al., 2023). In this context, our transfer learning framework facilitates the detection of both shared and species-specific parasite adaptation strategies across different *Leishmania* species with high predictive performance, as evaluated against temporally proximal transcriptomic states, with the closest match observed at 72hpi corresponding to patterns seen at 96hpi.

In addition, we investigated the functional relevance of three highly upregulated DEGs of unknown function that were consistently elevated in both predicted and experimentally derived datasets. Protein-peptide interaction and epitope prediction analyses identified several candidate parasite peptides with strong predicted binding affinity to MHC class II molecules, suggesting a potential role in host immune recognition. Future studies are warranted to experimentally validate these targets and further elucidate their roles in host-parasite interactions, thereby strengthening the translational potential of these findings. Importantly, these insights further emphasize the value of incorporating transfer learning approaches, as it can be adaptable to other *Leishmania* species and appears to capture key virulence-associated transcriptomic signatures, including those distinguishing VL from CL.

The temporal parasite transcriptional profile observed here is consistent with the well-established transition from promastigotes to intracellular amastigotes, which occurs within approximately 6–24 h post-infection and is followed by the transcriptomic transformation into immune-modulation and host-adapted state (Alvar et al., 2012). At 96hpi, both *L. infantum* and *L. amazonensis* exhibit signatures indicative of intracellular persistence, including upregulation of amastin-associated surface proteins and selective metabolic and stress-adaptation responses, alongside reduced representation of global translational machinery (**Figure 5**) (Fernandes et al., 2016). In *L. infantum*, the relative upregulation of GP63 (**Figure 5**) is known to contribute to parasite-favored immune-modulation, as GP63 has been shown to interfere with macrophage signaling and reduce NO-mediated parasite killing, a mechanism reported across VL systems (Olivier et al., 2005; Isnard et al., 2012). In contrast, *L. amazonensis* does not exhibit relative increase on GP63 expression at later stages, consistent with evidence that some of these species display relative resistance to NO-mediated leishmanicidal activity and may adopt an alternative survival strategy (Giudice et al., 2007). Notably, *L. amazonensis* shows enrichment of autophagy-related components, in agreement with previously reported ATG8-dependent pathways implicated in parasite differentiation and pathogenicity. Autophagy has been shown to be essential for mitochondrial homeostasis and infectivity in *L. major*, while in *L. amazonensis*, host-parasite interactions seem to actively engage autophagic processes to enhance intracellular survival (Sacks and Noben-Trauth, 2002; Olivier et al., 2005). This suggests that late-stage infection is characterized by a coordinated and conserved shift toward an autophagy-supported response that may promote parasite persistence and CL pathogenesis by various species.

A limitation of this study is the limited availability of publicly accessible RNA-seq datasets generated under comparable host-parasite infection conditions and matched-time points, resulting in a relatively small training dataset for the transfer learning framework. Although several strategies were implemented here to improve generalizability and reduce overfitting, including ortholog-based feature mapping across all species, multi-species training, and training-data augmentation, future transcriptomic datasets will be required in the future to further improve model prediction and reproducibility. Furthermore, validation of the predicted *L. amazonensis* 96hpi expression profiles was performed using the closest available independent dataset (72hpi) generated from primary human macrophages rather than THP-1-derived macrophages. Differences in host cell type and infection dynamics may therefore contribute to variability between predicted and observed expression profiles. Future *in vitro* studies incorporating harmonized infection models and additional transcriptomic datasets will enable more rigorous validation and further refinement of the proposed framework.

## 5 Conclusion

This study proposes an integrative framework for investigating host-parasite interactions in *Leishmania* infection by combining a systematic review, transcriptomic meta-analysis using a harmonized bioinformatic pipeline with batch correction, and a hypothesis-driven exploratory transfer learning approach. We demonstrate that THP-1-derived macrophage responses follow a temporal trajectory from early inflammatory activation to later immune regulation. Comparative analyses across *Leishmania* species revealed both conserved and species-specific host transcriptional responses. Furthermore, our hypothesis-driven transfer learning approach demonstrated the feasibility of predicting late-stage parasite gene expression in data-limited settings, while the identification of candidate antigenic peptides provides potential targets for future experimental validation. Overall, this work highlights the value of integrating multi-study transcriptomic datasets to advance our understanding of host-parasite interactions in leishmaniasis. It also presents an exploratory computational approach that leverages transcriptomic data from closely related *Leishmania* species to generate biologically testable hypotheses, particularly in neglected tropical disease research, where comparable and harmonized RNA-seq datasets are often scarce.

## Data Availability

The original contributions presented in the study are included in the article/**Supplementary Material**. Further inquiries can be directed to the corresponding author.

## Supplementary material

Supplementary Figure 1 PRISMA flow diagram of study selection for transcriptomic meta-analysis. Flow diagram illustrating the identification, screening, eligibility assessment, and inclusion of studies used in this meta-analysis, following PRISMA 2020 guidelines. A total of 19 records were initially identified from PubMed, with 2 excluded based on publication year. Seventeen articles were screened by title and abstract, of which several were excluded due to study type (reviews), non-transcriptomic approaches (proteomics, microarray/qPCR), or unsuitable experimental design. Five studies underwent full-text assessment, with additional exclusions applied due to duplicate use of sequencing datasets. Ultimately, three studies comprising 27 RNA-sequencing datasets were included in the final analysis, alongside one additional dataset used to support machine-learning model development.

Supplementary Figure 2 Principal component analysis (PCA) of the uncorrected combined transcriptomic dataset prior to batch correction. Principal component analysis (PCA) of the uncorrected combined transcriptomic dataset prior to batch correction. Library/study labels correspond to A = m4 = Gatto et al. (2020); B = m5 = Fernandes et al. (2023); and C = m6 = Perea-Martínez et al. (2022), while different colors denote the experimental conditions within each study (infected and control at the corresponding time points). Prior to batch correction, samples clustered predominantly according to study rather than biological condition, indicating the presence of substantial batch effects.

Supplementary Table 1 Consolidated physicochemical properties of the four peptides.

Supplementary Table 2 Per-complex interface summary.

Supplementary Table 3 Summary of protein-peptide docking interactions for all four complexes.
