# Supplementary material for: Integrative transcriptomics and hypothesis-driven transfer machine learning reveal conserved and species-specific host-parasite dynamics across Leishmania species in THP-1 cells: a systematic review and meta-analysis
Source: Front Cell Infect Microbiol. 2026 Aug 26;16:1891986. doi: 10.3389/fcimb.2026.1891986 (PMC13553918; doi:10.3389/fcimb.2026.1891986)
Supplement: Supplementary file 1 [file SupplementaryFile1.docx]

**Identification of studies via databases and registers**

Records removed for year of publication (n = 2)

Records identified from*:

PubMed (n=19)

**Identification**

Reports excluded:

Reviews (n=1)

proteomics (n= 1)

Microarray and qPCR (n=3)

Experiment design (n =7)

Articles screened based on title and abstract (n= 17)

**Screening**

Articles assessed for eligibility, full-text screening (n= 5)

Reports excluded:

Articles used already included sequencing files (n= 2)

Studies included to improve Machine-learning models (n= 1)

Raw sequencing files

(n= 11-21)

This includes *L. amazonensis-monocyte derived human macrophages* (24hpi, 72hpi) and *L. major-monocyte derived human macrophages* (24hpi, 72hpi).

Studies included in review

(n= 3)

Raw sequencing files

(n= 27)

This includes *L. infantum-THP-1* (8hpi, 24hpi, 96hpi) and *L. amazonensis-THP-1* (24hpi).

**Included**

Source: Page MJ, et al. BMJ 2021;372:n71. doi: 10.1136/bmj.n71.

This work is licensed under CC BY 4.0. To view a copy of this license, visit <https://creativecommons.org/licenses/by/4.0/>

**Supplementary Figure 1: PRISMA flow diagram of study selection for transcriptomic meta-analysis**

Flow diagram illustrating the identification, screening, eligibility assessment, and inclusion of studies used in this meta-analysis, following PRISMA 2020 guidelines. A total of 19 records were initially identified from PubMed, with 2 excluded based on publication year. Seventeen articles were screened by title and abstract, of which several were excluded due to study type (reviews), non-transcriptomic approaches (proteomics, microarray/qPCR), or unsuitable experimental design. Five studies underwent full-text assessment, with additional exclusions applied due to duplicate use of sequencing datasets. Ultimately, three studies comprising 27 RNA-sequencing datasets were included in the final analysis, alongside one additional dataset used to support machine-learning model development.
