# Supplementary material for: Integrative transcriptomics and hypothesis-driven transfer machine learning reveal conserved and species-specific host-parasite dynamics across Leishmania species in THP-1 cells: a systematic review and meta-analysis
Source: Front Cell Infect Microbiol. 2026 Aug 26;16:1891986. doi: 10.3389/fcimb.2026.1891986 (PMC13553918; doi:10.3389/fcimb.2026.1891986)
Supplement: Supplementary file 2 [file SupplementaryFile3.docx]

**Supplementary Table 1: Consolidated physicochemical properties of the four peptides.**

| **Property** | **Pep3_Prot3** | **Pep4_Prot1** | **Pep5_Prot1** | **Pep6_Prot2** |
| --- | --- | --- | --- | --- |
| **Molecular Weight** | ~1383 Da | ~1843 Da | ~1063 Da | ~1509 Da |
| **GRAVY Score** | +0.987 | −0.547 | +0.127 | +0.793 |
| **Isoelectric Point (pI)** | ~5.52 | ~8.75 | ~5.52 | ~5.59 |
| **Net Charge (pH 7)** | −1 | +2 | 0 | 0 |
| **Instability Index** | Stable (<40) | Borderline (~42) | Stable (<40) | Stable (<40) |
| **Aliphatic Index** | 127.3 | 71.3 | 68.0 | 130.7 |
| **Key Motif** | V8–V9–V10 (VVV) | K2–V5 & S8–R11 helices | P1–x–T3–P4 (PXTP) | V1–V2–A3–F4–V5 |
| **Dominant SS** | β-Sheet (47%) | α-Helix (67%) | Coil/IDR (60%) | β-Sheet + Helix |
| **Aromaticity** | F×1 | H×1, Y×1 | None | F×1, Y×1 |
| **Biological Character** | Hydrophobic / Membrane | Soluble / Basic | Linker / IDR | TM / Hydrophobic |
| **Predicted Function** | Membrane association / hydrophobic burial | DNA/RNA binding; cell-penetrating peptide | IDR / mucin O-glycosylation linker | Transmembrane / amphipathic helix |
