# Supplementary material for: Integrative transcriptomics and hypothesis-driven transfer machine learning reveal conserved and species-specific host-parasite dynamics across Leishmania species in THP-1 cells: a systematic review and meta-analysis
Source: Front Cell Infect Microbiol. 2026 Aug 26;16:1891986. doi: 10.3389/fcimb.2026.1891986 (PMC13553918; doi:10.3389/fcimb.2026.1891986)
Supplement: Supplementary file 3 [file SupplementaryFile4.docx]

**Supplementary Table 2: Per-complex interface summary:**

| **Structure** | **H-Bonds** | **Salt Bridges** | **Hydrophobic** | **Total** |
| --- | --- | --- | --- | --- |
| 1BX2_Pep4_Prot1 (HLA-DR2) | 68 | 6 | 42 | **116** |
| 1AQD_Pep6_Prot2 (HLA-DR1) | 65 | 3 | 43 | **111** |
| 4D8P_Pep5_Prot1 (HLA-DQ2.3) | 69 | 1 | 48 | **118** |
| 4D8P_Pep3_Prot3 (HLA-DQ2.3) | 71 | 2 | 46 | **119** |
