# Supplementary material for: Integrative transcriptomics and hypothesis-driven transfer machine learning reveal conserved and species-specific host-parasite dynamics across Leishmania species in THP-1 cells: a systematic review and meta-analysis
Source: Front Cell Infect Microbiol. 2026 Aug 26;16:1891986. doi: 10.3389/fcimb.2026.1891986 (PMC13553918; doi:10.3389/fcimb.2026.1891986)
Supplement: Supplementary file 4 [file SupplementaryFile5.docx]

**Supplementary Table 3: Summary of protein-peptide docking interactions for all four complexes.**

| **Protein-Peptide Complex** | **Bond Type** | **Count** | **Distance Range (Å)** | **Character** |
| --- | --- | --- | --- | --- |
| 1AQD_Pep6_Prot2 | H-Bonds | 19 | 2.61–3.44 | Polar electrostatic |
|  | Salt Bridges | 8 | 2.61–3.87 | Ionic / charged |
|  | Hydrophobic | 15 | 4.0–4.96 | Van der Waals / apolar |
|  | π–π Stacking | 11 | 4.14–4.94 | Aromatic ring stacking |
| 4D8P_Pep5_Prot1 | H-Bonds | 41 | 2.64–3.45 | Polar electrostatic |
|  | Salt Bridges | 7 | 3.55–3.98 | Ionic / charged |
|  | Hydrophobic | 40 | 3.68–4.99 | Van der Waals / apolar |
|  | π–π Stacking | 6 | 4.57–4.96 | Aromatic ring stacking |
| 4D8P_Pep3_Prot3 | H-Bonds | 41 | 2.54–3.45 | Polar electrostatic |
|  | Salt Bridges | 8 | 3.57–3.98 | Ionic / charged |
|  | Hydrophobic | 34 | 3.69–4.99 | Van der Waals / apolar |
|  | π–π Stacking | 6 | 4.31–4.96 | Aromatic ring stacking |
| 1BX2_Pep4_Prot1 | H-Bonds | 42 | 2.47-3.50 | Polar electrostatic |
|  | Salt Bridges | 11 | 2.47-3.99 | Ionic / charged |
|  | Hydrophobic | 35 | 3.35-4.50 | Van der Waals / apolar |
|  | π–π Stacking | -- |  | Not detected (<5  cutoff) |
